# Impact of New-Onset Persistent Left Bundle Branch Block on Reverse Cardiac Remodeling and Clinical Outcomes After Transcatheter Aortic Valve Replacement

**DOI:** 10.3389/fcvm.2022.893878

**Published:** 2022-05-27

**Authors:** Kyu Kim, Young-Guk Ko, Chi Young Shim, JiWung Ryu, Yong-Joon Lee, Jiwon Seo, Seung-Jun Lee, Iksung Cho, Sung-Jin Hong, Chul-Min Ahn, Jung-Sun Kim, Byeong-Keuk Kim, Geu-Ru Hong, Jong-Won Ha, Donghoon Choi, Myeong-Ki Hong

**Affiliations:** ^1^Division of Cardiology, Severance Cardiovascular Hospital, Yonsei University College of Medicine, Seoul, South Korea; ^2^Division of Cardiology, Yongin Severance Hospital, Yonsei University College of Medicine, Yongin, South Korea

**Keywords:** transcatheter aortic valve replacement, left bundle branch block, cardiac remodeling, prognosis, aortic stenosis

## Abstract

**Background:**

The clinical implication of new-onset left bundle branch block (LBBB) after transcatheter aortic valve replacement (TAVR) remains controversial. We investigated the impact of new-onset persistent LBBB on reverse cardiac remodeling and clinical outcomes after TAVR.

**Methods:**

Among 478 patients who had undergone TAVR for symptomatic severe aortic stenosis from 2011 to 2021, we analyzed 364 patients after excluding patients with pre-existing intraventricular conduction disturbance or a pacing rhythm before or during the indexed hospitalization for TAVR. Echocardiographic variables of cardiac remodeling at baseline and 1 year after TAVR were comprehensively analyzed. The primary outcome was a composite of cardiovascular death and hospitalization for heart failure. Secondary outcomes were all-cause death and individual components of the primary outcome.

**Result:**

New-onset persistent LBBB occurred in 41 (11.3%) patients after TAVR. The no LBBB group showed a significant increase in the left ventricular (LV) ejection fraction and decreases in LV dimensions, the left atrial volume index, and LV mass index 1 year after TAVR (all *p* < 0.001). However, the new LBBB group showed no significant changes in these parameters. During a median follow-up of 18.1 months, the new LBBB group experienced a higher incidence of primary outcomes [hazard ratio (HR): 5.03; 95% confidence interval (CI): 2.60–9.73; *p* < 0.001] and all-cause death (HR: 2.80; 95% CI: 1.38–5.69; *p* = 0.003). The data were similar after multivariable regression analysis.

**Conclusion:**

New-onset persistent LBBB after TAVR is associated with insufficient reverse cardiac remodeling and increased adverse clinical events.

## Introduction

Transcatheter aortic valve replacement (TAVR) is an effective alternative to surgical aortic valve replacement (SAVR) for symptomatic severe aortic stenosis (AS) in patients with high, intermediate, or low surgical risk (1–5). However, conduction disturbances and the subsequent need for a permanent pacemaker (PPM) implantation occur more frequently after TAVR than after SAVR and remain the main complications of TAVR (6). The development of periprocedural conduction disturbances during TAVR is caused by direct mechanical insult to the conduction system, located in the proximity of the aortic valve (6). New-onset left bundle branch block (LBBB) is the most common conduction disturbance following TAVR, and its incidence varies from 4 to 30% with a balloon-expandable valve and 18–65% with a self-expandable valve (6). Despite its frequent incidence, the clinical implications of new-onset persistent LBBB after TAVR remain controversial (6–9). Several studies have reported conflicting results regarding the association between new-onset LBBB and increased cardiovascular mortality (7–10). Furthermore, limited data are available on the impact of new-onset LBBB on cardiac remodeling and function after TAVR. Because the indication for TAVR gradually expands to low-risk and younger patients, clarifying the true implication of new-onset LBBB following TAVR is crucial. Thus, the present study aimed to investigate the clinical impact of new-onset LBBB after TAVR on clinical outcomes and cardiac remodeling.

## Materials and Methods

### Study Population

A total of 478 consecutive patients who had undergone TAVR for symptomatic severe AS at Severance Cardiovascular Hospital from June 2011 to May 2021 were retrospectively reviewed. We excluded patients with pre-existing intraventricular conduction disturbances (*n* = 77) (QRS >120 ms, LBBB, and right bundle branch block) before TAVR and patients who had received permanent pacemaker implantation before or during the index hospitalization for TAVR (*n* = 34). There was no patient with previously implanted intracardiac cardioverter-defibrillator or cardiac resynchronization therapy. The present study also excluded three patients who did not survive immediately after TAVR. Thus, 364 patients were included in the final analysis in this study.

Surgical risk was estimated using the European System for Cardiac Operative Risk evaluation (EuroSCORE II) and the Society of Thoracic Surgeons Predicted Risk of Mortality (STS-PROM) score (11, 12). The decision to use TAVR as the treatment modality was made by a multidisciplinary heart team, as previously reported (13, 14). The transcatheter aortic valve type was chosen at the discretion of the operators based on the anatomical characteristics of the aortic valve, aortic root, and vascular access. The Institutional Review Board of Severance Hospital approved this study, which was conducted in compliance with the Declaration of Helsinki. The requirement for informed consent was waived because of the retrospective study design.

LBBB was defined as a QRS duration > 120 ms, delayed onset of intrinsicoid deflection in leads V5 and V6, broad monophasic R waves that are usually notched in leads I, V5 and V6, and secondary ST- and T-wave changes opposite in direction to the major QRS deflection (15). In this study, new-onset LBBB was defined as persistent LBBB developed during or after the TAVR procedure and documented on the electrocardiogram (ECG) at hospital discharge or 7 days after TAVR.

### Echocardiography

All echocardiographic studies were performed using commercially available equipment and were reviewed by imaging cardiologists without knowledge of the clinical data. Standard measurements were performed according to current guidelines (16). LV EF was measured using linear measurement or biplane methods. The LV mass index was calculated using the Devereux formula. The left atrial volume index (LAVI) was calculated using the biplane method. Pulmonary artery systolic pressure and right atrial pressure were estimated using tricuspid regurgitation jet velocity and inferior vena cava (16). Right ventricular (RV) systolic dysfunction was defined as in case of tricuspid annular plane systolic excursion <17 mm, tricuspid pulsed Doppler S wave <9.5 cm/s, or fractional area change <35% (16). Preprocedural and postprocedural AV hemodynamic parameters such as the AV peak flow velocity, transaortic pressure gradient, and aortic valve area were calculated using Doppler echocardiography (17). Concomitant at least moderate mitral or tricuspid regurgitation was defined as other valve pathology. The severity of paravalvular regurgitation was semi-quantitatively assessed according to recent recommendations (17). Patients underwent baseline echocardiography before TAVR and regular planned examinations annually after TAVR, according to standard institutional follow-up protocol. To investigate the impact of LBBB on reverse cardiac remodeling, baseline and 1-year echocardiographic parameters were compared according to the presence of LBBB.

### Clinical Outcomes

The primary outcome was a composite of cardiovascular death or hospitalization for heart failure. Secondary outcomes included all-cause death, cardiovascular death, hospitalization for heart failure, number of hospitalizations for heart failure event, and permanent pacemaker implantation. All clinical outcomes were analyzed according to the Valve Academic Research Consortium-3 consensus (18).

### Statistical Analysis

Categorical variables were presented as numbers (percentages) and were compared using chi-squared test or Fisher's exact test. Continuous variables were presented as means ± standard deviation and compared using Student's *t*-test or the Wilcoxon rank-sum test. Time-to-event variables were presented as Kaplan-Meier event rates and were compared using the log-rank test. The total number of hospitalizations for heart failure was calculated and compared using Poisson regression. Multivariable analysis for clinical outcomes was performed using a multivariable Cox proportional hazard regression model. The covariates included in the adjusted models were variables with clinical relevance, such as age, sex, New York Heart failure Association (NYHA) functional class, comorbidities such as chronic lung disease, end-stage renal disease, coronary artery disease, peripheral artery disease, prior cardiac surgery, atrial fibrillation, EuroSCORE II, STS-PROM score, baseline LV EF, RV systolic dysfunction, pulmonary artery systolic pressure and moderate or severe paravalvular regurgitation. The baseline and 1-year echocardiographic parameters were compared using paired *t*-test or the Wilcoxon signed-rank test as appropriate. Two-way repeated ANOVA was used to determine differences between the baseline and 1-year echocardiographic parameters according to the study groups. Missing data of 1-year echocardiographic data was not imputated. As sensitivity analysis, baseline and 1-year echocardiographic data was compared with multiple imputation of missing data. All the tests were two-tailed, and *p*-values <0.05 were considered statistically significant. All statistical analyses were performed using R version 4.1.0 (The R Foundation for Statistical Computing; www.R-project.org).

## Results

### Baseline Characteristics

Of the 364 patients, 41 (11.3%) had new-onset persistent LBBB after TAVR. The baseline clinical characteristics are shown in Table 1. The two groups, new LBBB group and no LBBB group, were similar in sex distribution, symptom severity, comorbidities, and the surgical risk score. The new LBBB group was younger than the no LBBB group, but the difference did not reach statistical significance. The new LBBB group had a higher prevalence of prior cardiac surgery. The choice of transcatheter aortic valve was not statistically different between the two groups; however, the new LBBB group had a trend toward more frequent use of self-expandable valves than the no LBBB group. Predilation rate was low in the new LBBB group, and the degree of paravalvular regurgitation was comparable in both groups.

**Table 1:** Baseline characteristics.

	**New LBBB** **(*N =* 41)**	**No LBBB** **(*N =* 323)**	***p*-value**
Age, years	79.7 ± 6.0	81.3 ± 5.3	0.080
Male sex, *n* (%)	17 (41.5)	149 (46.1)	0.690
NYHA class III-IV, *n* (%)	25 (61.0)	186 (57.6)	0.805
Hypertension, *n* (%)	37 (90.2)	268 (83.0)	0.334
Diabetes mellitus, *n* (%)	20 (48.8)	131 (40.6)	0.402
End-stage renal disease	5 (12.2)	21 (6.5)	0.312
Chronic lung disease	7 (17.1)	51 (15.8)	0.999
Cerebrovascular accident	8 (19.5)	45 (13.9)	0.472
Coronary artery disease	24 (58.5)	175 (54.2)	0.718
Previous myocardial infarction	5 (12.2)	24 (7.4)	0.450
Prior coronary intervention	11 (26.8)	75 (23.2)	0.751
Prior cardiac surgery	6 (14.6)	17 (5.3)	0.047
Coronary artery bypass	5 (12.2)	14 (4.3)	0.079
Mitral valve surgery	1 (2.4)	3 (0.9)	0.937
Atrial fibrillation	7 (17.1)	50 (15.5)	0.971
Peripheral artery disease	9 (22.0)	38 (11.8)	0.113
Concomitant other valve pathology	1 (2.4)	33 (10.2)	0.184
Mitral regurgitation	0 (0.0)	18 (5.6)	0.243
Tricuspid regurgitation	1 (2.4)	18 (5.6)	0.633
EuroSCORE II	4.7 ± 4.2	5.2 ± 8.0	0.474
STS-PROM, %	6.1 ± 5.3	5.8 ± 5.9	0.731
Valve			0.137
Corevalve	9 (22.0)	38 (11.8)	
Evolut Pro	5 (12.2)	52 (16.1)	
Evolut R	16 (39.0)	112 (34.7)	
LOTUS	2 (4.9)	6 (1.9)	
Sapien3	9 (22.0)	115 (35.6)	
Valve type			0.118
Balloon-expandable	9 (22.0)	115 (35.6)	
Self-expandable	32 (78.0)	208 (64.4)	
Predilatation	13 (31.7)	178 (55.1)	0.008
Postdilatation	10 (24.4)	119 (36.8)	0.162
Paravalvular regurgitation			0.878
No, trace	25 (61.0)	210 (65.0)	
Mild	13 (31.7)	92 (28.5)	
Moderate	3 (7.3)	21 (6.5)	

*LBBB, left bundle branch block; NYHA, New York Heart Association*.

### New-Onset LBBB and Reverse Cardiac Remodeling

The echocardiographic characteristics are summarized in Table 2. The baseline echocardiographic parameters were comparable between the new LBBB and no LBBB groups. One-year follow-up echocardiograms were obtained in 264 (73%) patients. At the 1-year follow-up after TAVR, both groups showed improved and similar AV hemodynamic parameters, such as the peak velocity, pressure gradient, and AV area; however, the new LBBB group showed a lower LV EF (59.1 ± 13.7 vs. 65.8 ± 9.6%; *p* = 0.018), a larger LV end-diastolic dimension (48.6 ± 5.5 vs. 46.5 ± 5.0 mm; *p* = 0.037) and end-systolic dimension (33.1 ± 6.7 vs. 30.3 ± 5.0 mm; *p* = 0.035), and higher E/e′ (28.2 ± 15.1 vs. 20.5 ± 7.9; *p* = 0.029) than the no LBBB group.

**Table 2:** Echocardiographic data.

	**New LBBB** **(*N =* 41)**	**No LBBB** **(*N =* 323)**	***p*-value**
Baseline, *n*	41 (100.0)	323 (100.0)	>0.999
LBBB at 30 days, *n*(%)	36/41 (87.9)	0/323 (0)	<0.001
AV peak velocity, m/s	4.2 ± 0.7	4.5 ± 0.7	0.007
AV mean pressure gradient, mmHg	43.8 ± 17.5	51.6 ± 17.2	0.008
AV area, cm^2^	0.8 ± 0.2	0.7 ± 0.2	0.123
Annulus diameter, mm	23.2 ± 2.5	23.5 ± 2.4	0.530
LV ejection fraction, %	61.9 ± 16.3	60.1 ± 14.2	0.461
LV end diastolic dimension, mm	48.2 ± 6.7	49.6 ± 6.4	0.187
Reduced LV ejection fraction (≤50%), *n* (%)	10 (24.4)	81 (25.1)	0.999
LV end systolic dimension, mm	32.6 ± 8.6	33.9 ± 7.5	0.310
LV mass index, g/m^2^	135.2 ± 35.0	144.7 ± 42.1	0.166
LA volume index, ml/m^2^	49.5 ± 14.0	52.3 ± 19.9	0.281
E/e′	22.3 ± 9.6	21.4 ± 9.0	0.565
Pulmonary artery systolic pressure, mmHg	36.8 ± 11.4	37.6 ± 13.8	0.735
Estimated right atrial pressure, mmHg	5.6 ± 1.7	6.3 ± 3.0	0.155
RV systolic dysfunction, *n* (%)	1 (2.4)	3 (0.9)	0.937
1-year follow up, *n*	28 (68.3)	236 (73.1)	0.646
LBBB at 1 year, *n* (%)	23/28 (82.1)	5/236 (2.1)	<0.001
AV peak velocity, m/s	2.2 ± 0.5	2.2 ± 0.5	0.882
AV mean pressure gradient, mmHg	10.3 ± 4.5	10.0 ± 4.7	0.830
Effective orifice area, cm^2^	1.7 ± 0.5	1.8 ± 0.4	0.187
LV ejection fraction, %	59.1 ± 13.7	65.8 ± 9.6	0.018
LV end diastolic dimension, mm	48.6 ± 5.5	46.5 ± 5.0	0.037
LV end systolic dimension, mm	33.1 ± 6.7	30.3 ± 5.0	0.035
LV mass index, g/m^2^	123.3 ± 29.5	119.2 ± 28.9	0.480
LA volume index, ml/m^2^	46.1 ± 16.7	45.3 ± 18.2	0.839
E/e′	28.2 ± 15.1	20.5 ± 7.9	0.029
Pulmonary artery systolic pressure, mmHg	32.8 ± 12.5	32.8 ± 9.8	0.994
Estimated right atrial pressure, mmHg	5.4 ± 1.4	5.5 ± 1.8	0.891
RV systolic dysfunction, *n* (%)	1/28 (3.6)	3/236 (1.3)	0.901

*AV, aortic valve; E/e′, ratio between the early mitral inflow velocity and mitral annular early diastolic velocity; LBBB, left bundle branch block; LA, left atrium; LV, left ventricle; RV, right ventricle*.

**Figure 1:**
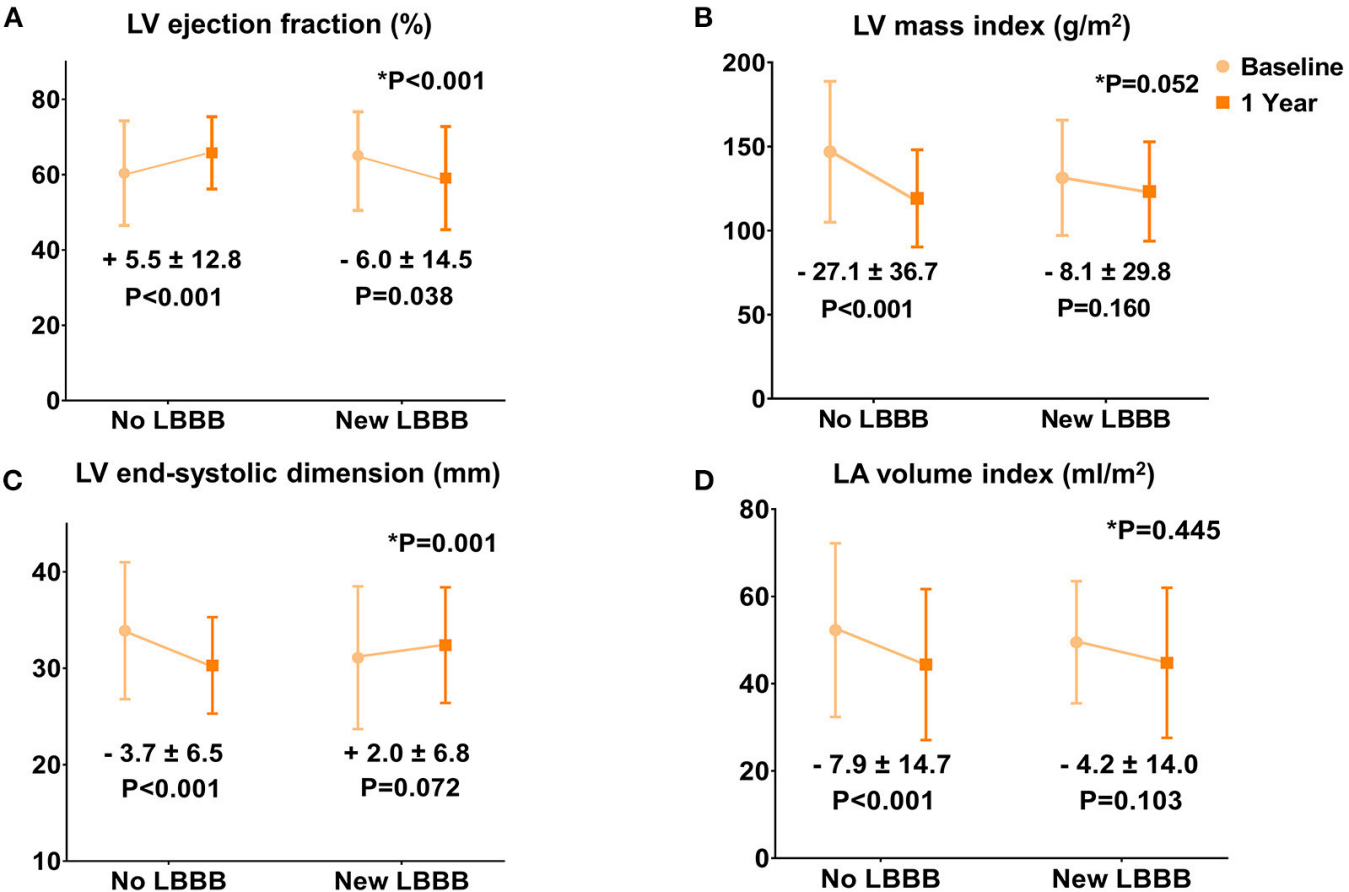
Changes in the echocardiographic parameters 1 year after TAVR. **(A)** Left ventricular ejection fraction. **(B)** Left ventricular mass index. **(C)** Left ventricular end-systolic dimension. **(D)** Left atrial volume index.

When the baseline and 1-year echocardiographic parameters were compared, the no LBBB group showed a significantly increased LV EF and a decreased LV end-systolic dimension, LV mass index, and left atrial (LA) volume index (all *p* < 0.001; Figure 1). However, the new LBBB group had significantly decreased LV EF (−6.0 ± 14.5%; *p* = 0.038) at the 1-year follow-up and no significant changes in the LV end-systolic dimension, LV mass index, and LA volume index. The sensitivity analysis with multiple imputation for 1-year echocardiographic data showed similar results (Supplementary Table 1 and Supplementary Figure 1). In the subgroup of patients with baseline LV systolic dysfunction (LV EF ≤ 50%), the new LBBB group showed no significant change in the LV EF (+8.2 ± 19.9%; *p* = 0.408), whereas the no LBBB group showed significant LV EF improvement (+20.5 ± 14.5%; *p* < 0.001) 1 year after TAVR. However, in patients with a preserved LV EF (>50%), the new LBBB group showed a significantly decreased LV EF (−9.0 ± 11.4%; *p* < 0.002) from the baseline while the no LBBB group had a similar LV EF (+1.0 ± 8.1%; *p* = 0.092) after TAVR (Supplementary Figure 2).

### New-Onset LBBB and Clinical Outcomes

**Figure 2:**
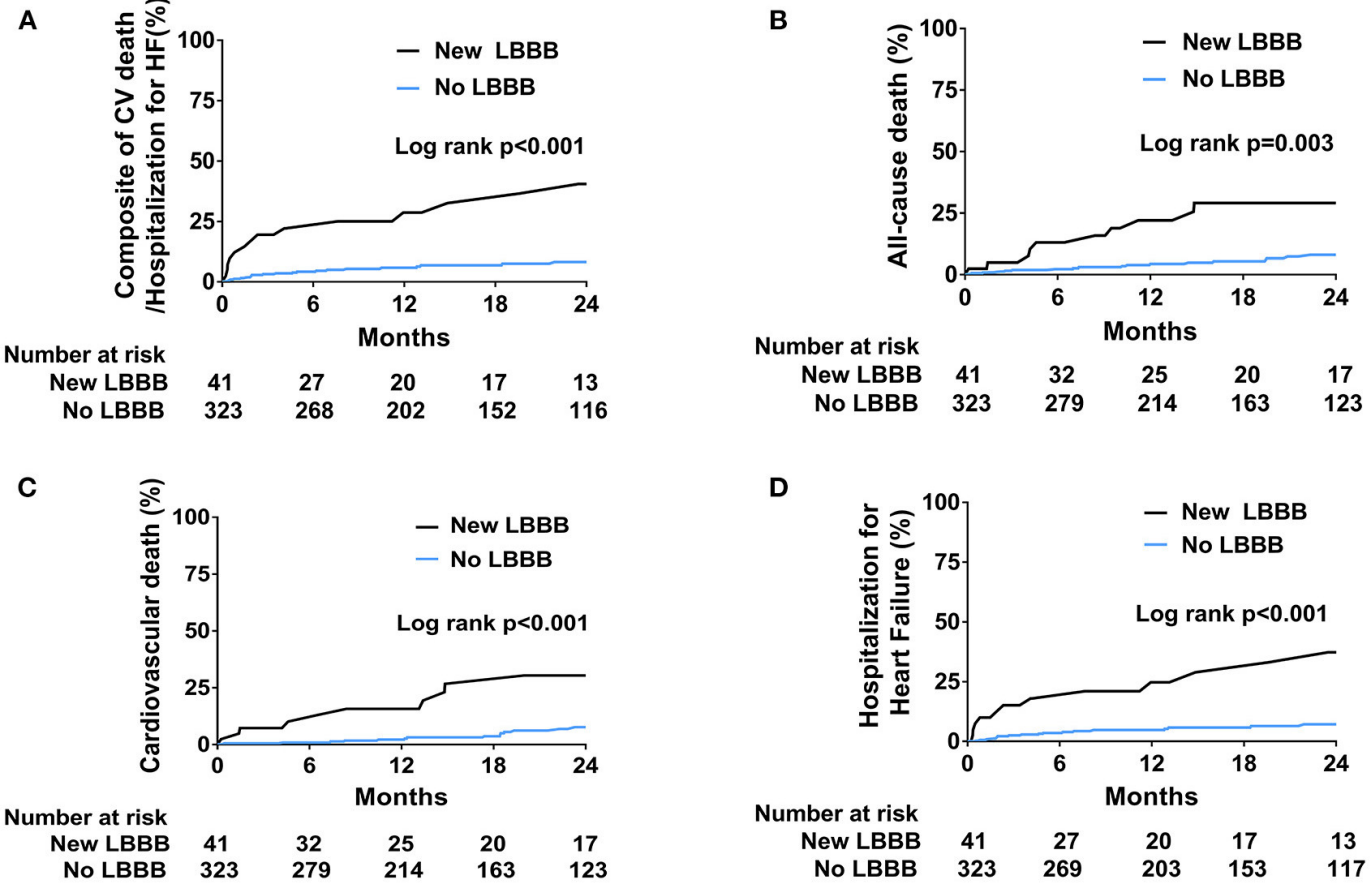
Kaplan-Meier curves for the incidence of clinical outcomes. **(A)** Composite of cardiovascular death and hospitalization for heart failure. **(B)** All-cause death. **(C)** Cardiovascular death. **(D)** Hospitalization for heart failure.

Patients were followed for a median of 18.1 months (interquartile range: 7.7–30.1). All the clinical outcomes after TAVR and hazard ratios for the adverse clinical events are described in Table 3 and Figure 2. The new LBBB group showed a higher rate of primary composite outcome events (cardiovascular death or hospitalization for heart failure) than the no LBBB group (HR: 5.03; 95% CI: 2.60–9.73; *p* < 0.001). The new LBBB group had a higher risk of all-cause death (HR: 2.80; 95% CI: 1.38–5.69; *p* = 0.003), individual events of cardiovascular death (HR: 7.34; 95% CI: 2.35–22.93; *p* < 0.001), and hospitalization for heart failure (HR: 5.25; 95% CI: 2.57–10.75; *p* < 0.001). Furthermore, the new LBBB group had more hospitalizations for heart failure (29.4 vs. 5.1 events per 100-person year; *p* < 0.001) and PPM implantation than the no LBBB group (HR: 5.44; 95% CI: 1.21–24.52; *p* = 0.010). There was no post-procedural CRT implantation. After multivariable adjustment, the patients with new-onset LBBB still had a significantly higher risk for adverse clinical outcomes, with the exception of PPM implantation. In Cox multivariate regression analysis, new-onset persistent LBBB, end-stage renal disease, atrial fibrillation, and prior caridac surgery were identified as independent predictors for the composite events of cardiovascular death and hospitalization for heart failure (Table 4).

**Table 3:** Clinical outcomes.

**Events, *n* (%/year)**	**New LBBB** **(*N =* 41)**	**No LBBB** **(*N =* 323)**	**Hazard ratio (95% CI),** ***p*****-value**	
			**Crude**	**Adjusted**
**Primary outcome**				
Cardiovascular death or hospitalization for heart failure	14 (18.9)	24 (4.2)	5.03 (2.60–9.73), *p <* 0.001	5.85 (2.87–11.95), *p <* 0.001
**Secondary outcomes**				
Cardiovascular death	6 (6.5)	6 (1.0)	7.34 (2.35–22.93), *p <* 0.001	7.79 (1.89–32.10), *p <* 0.001
Hospitalization for heart failure	12 (16.2)	20 (3.5)	5.25 (2.57–10.75), *p <* 0.001	5.21 (2.49–10.94), *p <* 0.001
All-cause death	11 (12.0)	27 (4.5)	2.80 (1.38–5.69), *p =* 0.003	2.47 (1.14–5.39), *p =* 0.023
Permanent pacemaker implantation	3 (3.3)	4 (0.7)	5.44 (1.21–24.5), *p =* 0.010	5.89 (0.91–38.23), *p =* 0.063
Number of heart failure hospitalization	27 (29.4)	31 (5.1)	5.91 (3.52–9.95), *p <* 0.001	5.25 (2.90–9.49), *p <* 0.001

*CI, confidence interval; LBBB, left bundle branch block*.

**Table 4:** Univariate and multivariable predictors of clinical outcomes after TAVR.

**Variables**	**Univariable**		**Multivariable**	
	**Hazard ratio (95% CI)**	***P*-value**	**Hazard ratio (95% CI)**	***p*-value**
New LBBB	5.03 (2.60–9.73)	<0.001	5.85 (2.87–11.95)	<0.001
Age, per year	0.95 (0.90–1.000)	0.050	1.00 (0.94–1.07)	0.941
Male sex	0.84 (0.44–1.60)	0.599	0.59 (0.27–1.25)	0.167
NYHA III-IV (vs. NYHA I-II)	1.23 (0.64–2.39)	0.533	1.24 (0.60–2.57)	0.566
Chronic lung disease	0.28 (0.07–1.17)	0.082	0.42 (0.10–1.87)	0.256
End stage renal disease	4.78 (2.18–10.47)	<0.001	5.93 (1.91–18.39)	0.002
Coronary artery disease	2.12 (1.05–4.28)	0.035	1.85 (0.86–3.99)	0.118
Peripheral artery disease	2.50 (1.22–5.15)	0.013	1.69 (0.72–3.95)	0.226
Prior cardiac surgery	3.77 (1.72–8.24)	<0.001	2.58 (1.04–6.39)	0.040
Atrial fibrillation	2.93 (1.50–5.73)	0.002	3.37 (1.57–7.25)	0.002
Euroscore II	1.02 (1.00–1.05)	0.079	0.97 (0.91–1.04)	0.429
STS-PROM	1.04 (1.01–1.07)	0.005	1.03 (0.96–1.10)	0.491
Baseline LV ejection fraction	0.97 (0.95–0.99)	<0.001	0.98 (0.95–1.01)	0.134
RV systolic dysfunction	4.64 (1.11–19.34)	<0.001	1.15 (0.22–6.01)	0.869
Pulmonary artery systolic pressure	1.03 (1.01–1.05)	0.002	1.03 (1.00–1.05)	0.080
Paravalvular regurgitation, moderate or severe	3.33 (1.39–8.00)	0.008	2.37 (0.86–6.48)	0.166

*LBBB, left bundle branch block; LV, left ventricle; RV, right ventricle*.

## Discussion

The major findings of the present study were as follows: 1) new-onset persistent LBBB occurred in 11.3% of patients without significant baseline conduction disturbances; 2) new-onset LBBB was associated with insufficient reverse cardiac remodeling and decline in the LV EF 1 year after TAVR; 3) new-onset LBBB was associated with increased occurrence of hospitalization for heart failure, cardiovascular death, and all-cause death.

The incidence of new-onset LBBB in previous studies varies widely because of differences in the inclusion of transient LBBB, timing of measurement, and type of transcatheter valve (6). Generally, new-onset LBBB occurs more frequently with self-expandable valves than with balloon-expanding valves (19). In the present study, the new LBBB group was also treated more frequently with self-expanding than balloon-expanding valves.

LBBB is associated with a shortening of LV diastole, abnormal septal motion with an associated decrease in the regional ejection fraction and an overall reduction in the global LV ejection fraction (20). LBBB further contributes to a vicious circle of LV wall stress, asymmetric hypertrophy, and dilatation that progressively deteriorates LV function (21).

Because concentric LV hypertrophy and reduced contractility is the main cardiac manifestation derived from pressure overload, the reversal of cardiac remodeling is a critical therapeutic target in patients with severe AS (22). However, in the present study, new-onset persistent LBBB after TAVR was associated with insufficient reverse cardiac remodeling and decreased LV function. Although patients without conduction abnormalities after TAVR showed increased LV EF and decreased LV and LA dimensions with improved diastolic function at the 1-year follow-up, the patients with new-onset LBBB revealed declined LV EF and no significant reduction in the LV and LA dimensions. Nazif et al. (8) also reported similar findings in a retrospective analysis from the PARTNER II trial. Patients with new LBBB after TAVR demonstrated a decline in the LV EF and increased LV dimensions at 1 and 2 years. Similarly, among patients who had undergone aortic valve surgery, those with electrical dyssynchrony, such as LBBB, and those with an electrical pacing rhythm showed no significant improvement in LV EF compared with patients without conduction disturbance (23).

LBBB is a significant risk factor for both cardiovascular and all-cause mortality in patients with various cardiovascular diseases (24). Houthuizen et al. (25) first demonstrated the association of new-onset LBBB with increased mortality after TAVR. However, further clinical studies did not confirm this association, and the clinical implication of new-onset LBBB after TAVR remains controversial (7–9). Recently, Nazif et al. (8) also reported that new-onset LBBB after TAVR increased the incidence of adverse clinical events such as all-cause and cardiovascular mortality, rehospitalization, and new pacemaker implantation. Additionally, a meta-analysis found an association between new LBBB and increased cardiovascular mortality (7). Our findings are consistent with those of these previous studies (7, 8). The discrepancy among study results regarding the association of LBBB with increased mortality and adverse clinical outcomes may be due to different definitions of LBBB in the different studies, characteristics of the study different populations, and variability in follow-up durations. The mechanism underlying the association of new LBBB after TAVR with a poor clinical prognosis remains unknown. An insufficient reversal of cardiac remodeling and decreased LV systolic function may contribute to increased incidences of hospitalization for heart failure and cardiovascular mortality. Because conduction disturbance is more frequently observed after TAVR than after SAVR, efforts must be made to reduce this complication before the TAVR indications are expanded to younger patients, who have a longer expected survival.

### Limitations

This study has several limitations. First, this study was a single-center retrospective study, which has inherent limitations. Second, the number of subjects with new LBBB was too small for detailed subgroup analysis. Third, this study included subjects with an advanced age and at variable surgical risk, possibly limiting the generalization of our study results to younger patients, who are at a lower surgical risk. Finally, 1-year follow-up echocardiography data were not available for all patients due to the early occurrence of clinical outcomes and the advanced age of the study population. However, we performed the multiple imputation for missing 1-year echocardiographic data and found similar results with main findings.

## Conclusion

New-onset persistent LBBB following TAVR is associated with insufficient reverse cardiac remodeling and increased adverse clinical events such as all-cause death, cardiovascular death, and hospitalization for heart failure.

## Data Availability Statement

The raw data supporting the conclusions of this article will be made available by the authors, without undue reservation.

## Ethics Statement

The studies involving human participants were reviewed and approved by Severance Hospital, Yonsei University College of Medicine. Written informed consent for participation was not required for this study in accordance with the national legislation and the institutional requirements.

## Author Contributions

KK and Y-GK contributed to the conception and design of the work. KK, Y-GK, and CS drafted the manuscript. JR, Y-JL, JS, S-JL, IC, S-JH, C-MA, J-SK, B-KK, and G-RH assisted in data collection and analysis. J-WH, DC, and M-KH contributed to the review and revision of the manuscript. All authors contributed to the article and approved the submitted version.

## Conflict of Interest

The authors declare that the research was conducted in the absence of any commercial or financial relationships that could be construed as a potential conflict of interest.

## Publisher's Note

All claims expressed in this article are solely those of the authors and do not necessarily represent those of their affiliated organizations, or those of the publisher, the editors and the reviewers. Any product that may be evaluated in this article, or claim that may be made by its manufacturer, is not guaranteed or endorsed by the publisher.

## Abbreviations

AS: Aortic stenosis
AV: Aortic valve
EF: Ejection fraction
LBBB: Left bundle branch block
LV: Left ventricle
NYHA: New York Heart Association
PPM: Permanent pacemaker
SAVR: Surgical aortic valve replacement
TAVR: Transcatheter aortic valve replacement.
