# Supplementary material for: Impact of New-Onset Persistent Left Bundle Branch Block on Reverse Cardiac Remodeling and Clinical Outcomes After Transcatheter Aortic Valve Replacement
Source: Front Cardiovasc Med. 2022 May 27;9:893878. doi: 10.3389/fcvm.2022.893878 (PMC9196075; doi:10.3389/fcvm.2022.893878)
Supplement: Supplementary file 1 [file Data_Sheet_1.docx]

**Supplementary Figure 1.** Changes in the echocardiographic parameters 1 year after TAVR with multiple imputation. (A) Left ventricular ejection fraction. (B) Left ventricular mass index. (C) Left ventricular end-systolic dimension. (D) Left atrial volume index.

**
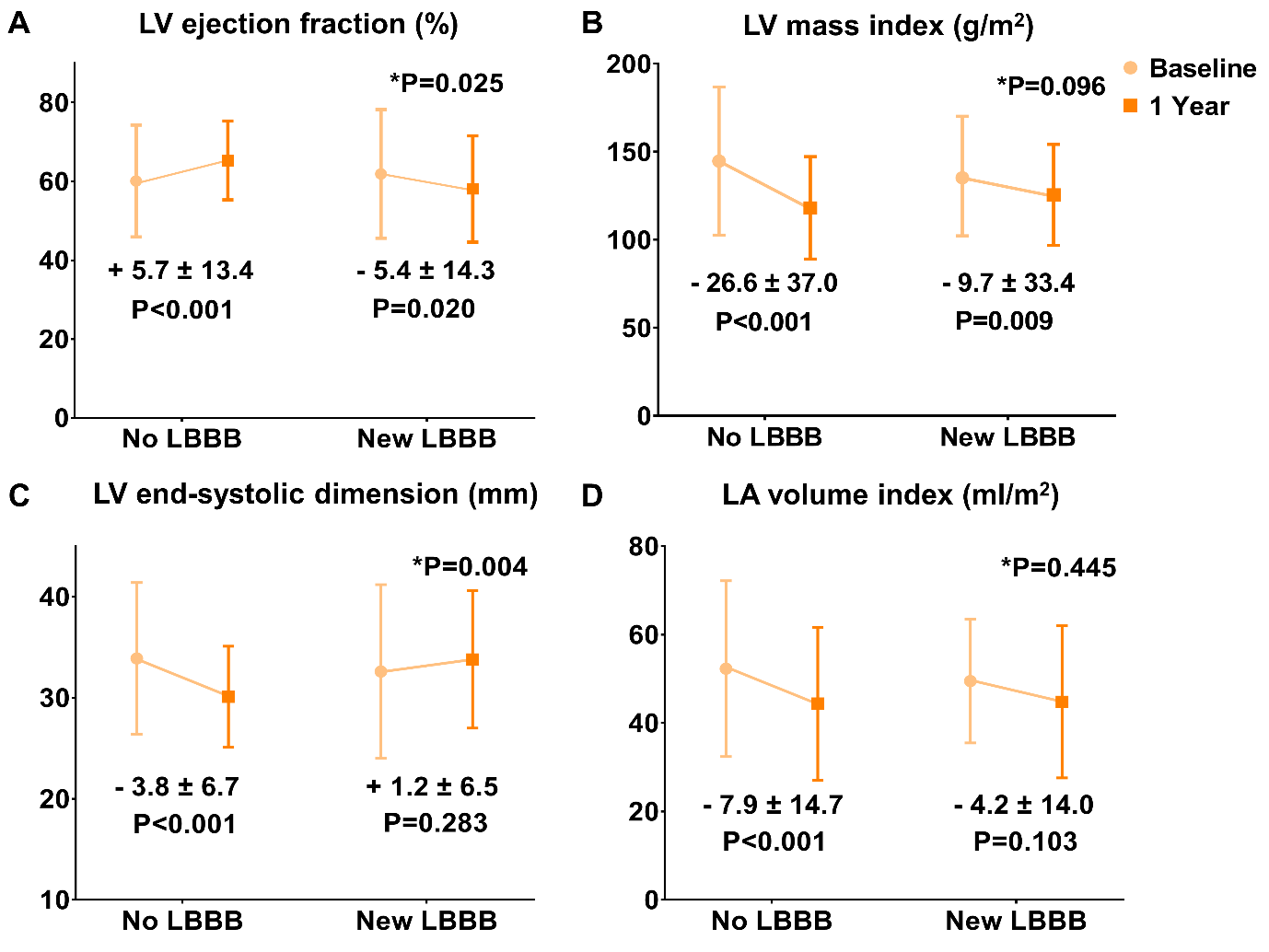
**
