# Supplementary material for: Impact of New-Onset Persistent Left Bundle Branch Block on Reverse Cardiac Remodeling and Clinical Outcomes After Transcatheter Aortic Valve Replacement
Source: Front Cardiovasc Med. 2022 May 27;9:893878. doi: 10.3389/fcvm.2022.893878 (PMC9196075; doi:10.3389/fcvm.2022.893878)
Supplement: Supplementary file 2 [file Data_Sheet_2.docx]

**Supplementary Figure 2.** Changes in the left ventricular ejection fraction 1 year after TAVR, according to the baseline left ventricular systolic function. (A) Overall study population. (B) Preserved ejection fraction (> 50%). (C) Reduced ejection fraction (≤ 50%).

**
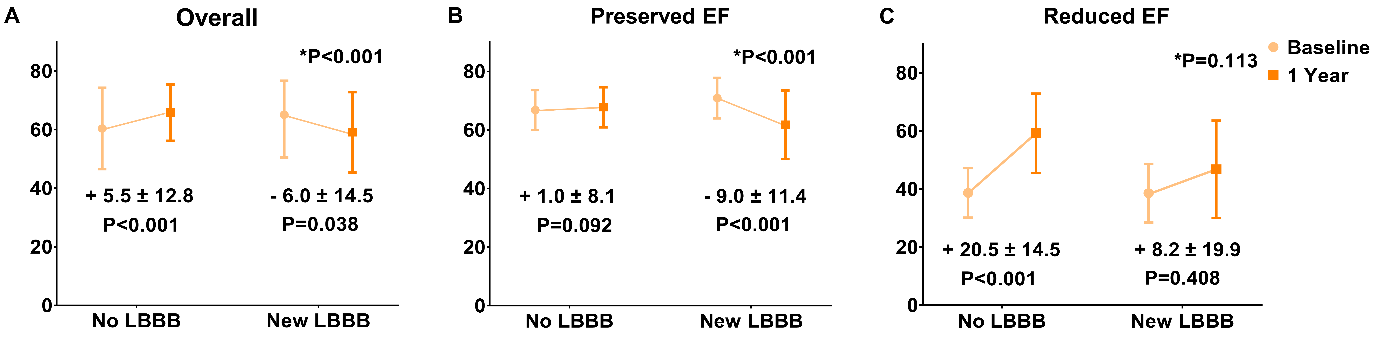
**

^*^P-value between the new LBBB and no LBBB group.
