# Supplementary material for: Impact of New-Onset Persistent Left Bundle Branch Block on Reverse Cardiac Remodeling and Clinical Outcomes After Transcatheter Aortic Valve Replacement
Source: Front Cardiovasc Med. 2022 May 27;9:893878. doi: 10.3389/fcvm.2022.893878 (PMC9196075; doi:10.3389/fcvm.2022.893878)
Supplement: Supplementary file 3 [file Table_1.DOCX]

**Supplementary Table 1. Echocardiographic data with multiple imputation**

|  | **New LBBB (N=41)** | **No LBBB (N=323)** | **p value** |
| --- | --- | --- | --- |
| Baseline, n | 41 (100.0) | 323 (100.0) | >0.999 |
| AV peak velocity, m/s | 4.2 ± 0.7 | 4.5 ± 0.7 | 0.007 |
| AV mean pressure gradient, mmHg | 43.8 ± 17.5 | 51.6 ± 17.2 | 0.008 |
| AV area, cm^2^ | 0.8 ± 0.2 | 0.7 ± 0.2 | 0.123 |
| Annulus diameter, mm | 23.2 ± 2.5 | 23.5 ± 2.4 | 0.530 |
| LV ejection fraction, % | 61.9 ± 16.3 | 60.1 ± 14.2 | 0.461 |
| LV end diastolic dimension, mm | 48.2 ± 6.7 | 49.6 ± 6.4 | 0.187 |
| Reduced LV ejection fraction (≤50%), n (%) | 10 (24.4) | 81 (25.1) | 0.999 |
| LV end systolic dimension, mm | 32.6 ± 8.6 | 33.9 ± 7.5 | 0.310 |
| LV mass index, g/m^2^ | 135.2 ± 35.0 | 144.7 ± 42.1 | 0.166 |
| LA volume index, ml/m^2^ | 49.5 ± 14.0 | 52.3 ± 19.9 | 0.281 |
| E/e` | 22.3 ± 9.6 | 21.4 ± 9.0 | 0.565 |
| 1 year follow up |  |  |  |
| AV peak velocity, m/s | 2.2 ± 0.4 | 2.1 ± 0.4 | 0.692 |
| AV mean pressure gradient, mmHg | 10.5 ± 4.5 | 10.3 ± 4.7 | 0.768 |
| Effective orifice area, cm^2^ | 1.7 ± 0.5 | 1.8 ± 0.4 | 0.170 |
| LV ejection fraction, % | 59.3 ± 13.3 | 65.7 ± 9.2 | 0.005 |
| LV end diastolic dimension, mm | 47.6 ± 5.4 | 46.7 ± 4.5 | 0.313 |
| LV end systolic dimension, mm | 32.7 ± 7.3 | 29.8 ± 4.6 | 0.018 |
| LV mass index, g/m2 | 124.5 ± 33.4 | 119.1 ± 28.8 | 0.330 |
| LA volume index, ml/m2 | 49.5 ± 19.0 | 44.8 ± 17.7 | 0.139 |
| E/e` | 27.1 ± 14.0 | 20.6 ± 8.8 | 0.005 |

Abbreviation; AV, aortic valve; IVS, interventricular septum; LBBB, left bundle branch block; LA, left atrium; LV, left ventricle; PW, posterior wall; E/e`, the ratio between early mitral inflow velocity and mitral annular early diastolic velocity
